# Mapping the genomic basis of developmental and metabolic responses to nitrogen

**DOI:** 10.1093/plcell/koac291

**Published:** 2022-09-27

**Authors:** Lucas Frungillo

**Affiliations:** Assistant Features Editor, The Plant Cell, American Society of Plant Biologists, USA; Institute of Molecular Plant Sciences, School of Biological Sciences, University of Edinburgh, UK

Nitrogen (N) is an essential macronutrient that regulates developmental and physiological responses in plants. Although phenotypic and metabolic responses to N in different forms and concentrations have been extensively reported (reviewed in Fredes et al., 2019), these studies have largely focused on the model plant *Arabidopsis thaliana* accession Columbia (Col-0), and how this information translates to natural variation in N responses is less understood. In this issue of *The Plant Cell*, **Ella Katz and colleagues (Katz et al., 2022)** use over a thousand accessions to map various new loci underpinning responses to different N regimens in Arabidopsis. Interestingly, the authors present evidence that combinations of many small effect loci, rather than a few large effect loci, modulate N responses, suggesting that synthetic manipulation of sets of genes is a promising strategy to improve N use efficiency in plants.

To investigate the phenotypic diversity of the responses to N source and availability, the authors optimized growth conditions under various nitrate and ammonium concentrations using the reference Arabidopsis accession Col-0. This strategy allowed them to identify the lowest and highest N concentrations in media that maximize trait variation in Arabidopsis with minimal developmental or growth penalties. The authors then took advantage of sequenced natural Arabidopsis genotypes from various geographical locations (The 1001 Genomes Consortium, 2016) to investigate responses to different N regimens. By testing response to N in 1,135 genotypes (Figure, left) while using a linear model to resolve the effect of genotype, growth condition, and interaction of both, the authors found that genotype exerted greater influence on the phenotypical trait variation. Additionally, despite some genotypes performing better when grown in ammonium, analysis of trait distribution for each N regimen revealed that the average Arabidopsis genotype displays higher biomass when cultivated on nitrate. Collectively, these data show that, although N source and availability significantly impacts developmental traits, phenotypic responses to N are mainly determined by the genotype.

**Figure:**
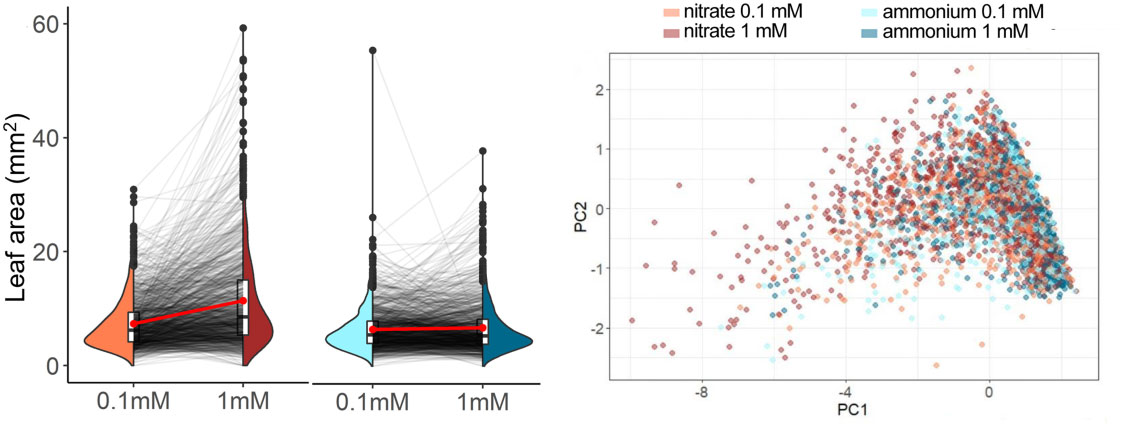
N regimens differentially affect developmental and metabolic traits across Arabidopsis natural accessions. A, Leaf area of 1,135 Arabidopsis ecotypes grown under different N regimens. B, Principal component analysis using developmental traits and color based on N condition. Adapted from Katz et al. (2022), Figure 2A (left) and Supplemental Figure S7A (right).

After N assimilation into organic compounds, the amino acids formed are used not just for protein synthesis but as substrates to produce a myriad of other secondary metabolites in plants. Because glucosinolates (GSLs) are a class of N-containing defense metabolites synthesized from different amino acids (reviewed in Yan and Chen, 2007), the authors hypothesized that overall GSL content represents a proxy for amino acid utilization in plants. Quantification of GSLs by high-performance liquid chromatography followed by linear modeling revealed that, reminiscent to phenotypic responses, interaction between genotype and nitrogen regimen underlies most GSLs variation. Additionally, to investigate links between developmental and metabolic responses to N regimen, the authors used a nonparametric correlation test to calculate genetic correlations between phenotypical traits and GSLs content in the Arabidopsis population. Separate correlation analyses for N source and concentration revealed that phenotypical traits are positively correlated to N regimen (Figure, right), while defense metabolites showed more diversity in their correlations. By using publicly available single-nucleotide polymorphism data combined with genome-wide association analysis, the authors provide a list of candidate genes associated with responses to N for the research community to investigate further.

In conclusion, Katz et al. (2022) elucidate genetic nodes controlling responses to N regime and show compelling evidence that these responses go far beyond those previously reported in Col-0. Their large-scale work provides a valuable resource for future genetic studies aimed at dissecting plant responses to N.
